# Replacement of threonine-55 with glycine decreases the reduction rate of OsTrx20 by glutathione

**Published:** 2017-03

**Authors:** Mitra Roodgar-Nashta, Azar Shahpiri

**Affiliations:** Department of Biotechnology, College of Agriculture, Isfahan University of ‎Technology, Isfahan, Iran.‎

**Keywords:** Glutathione, NADPH-dependent thioredoxin reductase, Rice, Thioredoxin

## Abstract

Thioredoxins (Trxs) are small ubiquitous oxidoreductase proteins with two redox-active Cys residues in a conserved active site (WCG/PPC) that regulate numerous target proteins via thiol/disulﬁde exchanges in the cells of prokaryotes and eukaryotes. The isoforms OsTrx23 with a typical active site (WCGPC) and OsTrx20 with an atypical active site (WCTPC) are two Trx h- type isoforms in rice that were previously found to be reduced by NADPH-dependent thioredoxin reductase and GSH/Grx system, respectively. In the present work the reduction of mutants G41T_OsTrx23_, T55G_OsTrx20_, K48D_OsTrx20_ and T55G-K48D _OsTrx20_ as well as wild types OsTrx23 and OsTrx20 were tested in the reaction containing either NADPH/NTR or glutathione (GSH). The results revealed that reduction rate of T55G_OsTrx20_ was remarkably decreased by GSH as compared to WtOsTrx20 highlighting the critical role of Thr-55 in interaction of OsTrx20 with GSH. On the other hand a significant decrease in the reduction rate of G41T_OsTrx23_ was observed in reaction containing NADPH-dependent thioredoxin reductase as compared with readuction rate of WtOsTrx23. These results suggest that first residue after N-terminal active site Cys is one of the critical residue in determination of system that Trxs can be reduced in.

## INTRODUCTION

Thioredoxins (Trxs) are small proteins (~12-14 kDa) with the highly conserved active site sequence motif, WCG(A/P)PC, and protein disulfide reductase activity [1]. Trxs act as electron donors to enzymes of metabolism such as ribonucleotide reductase, and play a critical role in the maintenance of reduced environment inside the cell [2]. In plants, Trxs are present in multiple forms based on primary structures and subcellular localization. Whereas Trxs f, m, x, and y are found in the chloroplast, Trxs o and h are localized to the cytoplasm or mitochondrion [3]. Trx f is reduced by ferredoxin/ thioredoxin reductase and in turn reduced by ferredoxin generated during photosynthetic electron transport [4]. In contrast, reduction of Trx o and two large Trx h subgroups (subgroups I and II) depends on NADPH and involves NADPH-dependent thioredoxin reductase (NTR) [3, 5]. In subgroup III, there are Trx h isoforms belonging to poplar and *Medicago truncatula* that have been previously reported to be reduced by a GSH-dependent pathway [6,7]. 

NTRs in bacteria, archaea, fungi and plants are homodimers of ~35 kDa subunits, each containing a NADP-binding domain and a FAD-binding domain [8, 9]. The NADP-binding domain of NTR contains two redox-active cysteines in a conserved active site sequence motif, CAV(T)C [10]. The cysteines form a disulfide bond in the oxidized NTR and receive electrons from NADPH via the coenzyme FAD. For the reduction of Trx, one of the two cysteine thiols in the reduced NTR attacks the active-site disulfide bond in the oxidized Trx, and an intermolecular disulfide bond is formed as a reaction intermediate[11]. Structural information of this intermediate is available in an engineered NTR-Trx complex from barley obtained at a resolution of 3.0 Å [12].

NTR-Trx interaction is highly specific, and commonly NTR from different species prefer Trx from the same species as substrate [13-15]. For instance Arabidopsis NTR (AtNTR) shows 80-fold higher affinity towards AtTrx than *E. coli* Trx (EcTrx) despite the high structural conservation between NTRs and Trxs from the two organisms [13]. Moreover, the affinity of HvNTR isoforms is 20 fold higher than that from AtNTR towards HvTrxh isoforms [16]. These incompatibilities between NTR and Trx from different sources show the high specificity in the interaction between NTR and Trx [16]. We have previously cloned and produced the recombinant form of two cytoplasmic/mitochondrial type NTR (OsNTRA and HvNTRB) [17]. and three rice Trx h isoforms (OsTrx1, OsTrx20 and OsTrx23) [18]. An extensive analysis on the reaction kinetic of the rice NTR/Trx system showed OsTrx1 and OsTrx23 were reduced by both OsNTR isoforms [19]. However, the isoform OsTrx20, a member of subgroup III of *h-type*, with an atypical active site _53_WCTPC_57_ was not reduced with NTR isoforms but instead it was reactivated by GSH/Grx system. Similarly MtTrx8 with WCSPC was not reduced by MtNTR isoforms [6, 19]. Another obvious difference between OsTrx20 with other plant Trx h isoforms was the presence of Lys-48 as a basic amino acid that has been occupied with Asp or Asn in the most of other plant Trx h isoforms.

In the present work, to understand the role of Thr55_OsTrx20_, Lys48_OsTrx20_ and Gly41_OsTrx23 _in reduction of Trxs by either NADPH/NTR system or GSH/glutaredoxin system the previously made mutants T55G_OsTrx20_, K48D_OsTrx20_, T55G-K48D_ OsTrx20_ and G41T_OsTrx23 _[20]. as well as the wild types WtOsTrx20 and WtOsTrx23 were heterologously expressed and purified as His.tag fusion protein and were used in reactions that either NADPH or GSH served as electron donor. 

## MATERIALS AND METHODS

**Heterogonous expression and purification of proteins: **The previously *E. coli* strains producing OsNTRB [17], WtOsTRx20, WtOsTrx23 [18], G41T_OsTrx23_, T55G_OsTrx20_, K48D_OsTrx20 _and T55G-K48DOsTrx20 [20]. were grown at 37℃ in LB medium supplemented with ampicillin (100 µg/ml) and chloramphenicol (5 ‎µ‎g/ml) to an OD_600_ of 0.6. Cultures were induced with 100 µM isopropyl β-d-thiogalactopyranoside (IPTG) for 4 h. The Cells were harvested by centrifugation and frozen at −80‎℃‎ until use. For extraction of soluble proteins, cells were resuspended in Tris–HCl 10 mM, pH 8. The cells were lysed by sonication with 70 % cycle and 100 % amplitude for 45 min at 4^º^C. After centrifugation (12,000 rpm for 20 min at 4‎℃‎), the extracted soluble proteins in the supernatant were transferred in new tubes. The purification of His-tegged recombinant protein was performed using HisTrap HP columns (GE Healthcare). HisTrap HP columns are prepacked with Ni Sepharose High Performance and designed for simple, high-resolution purification of histidine-tagged proteins by immobilized metal ion affinity chromatography (IMAC). The extracted soluble proteins was applied onto His-Trap HP columns preequilibrated with loading buffer (10 mM imidazole, 500 mM NaCl, 30 mM Tris–HCl [pH 8.0]) and eluted in a gradient of 10–50 % imidazole. Eluted fractions were analyzed by the SDS–PAGE procedure. Puriﬁed proteins were desalted in 10 mM Tris–HCl buffer (pH 8.0) at 4℃ using 12 kDa molecular weight cut-off cellulose acetate membranes (Sigma-Aldrich). The concentration of proteins were determined by A_280_ and Beer–Lambert law using molar extinction coefﬁcient 17085 M^−1^ cm^−1^ for WtOsTrx20, T55G_OsTrx20_, K48D_OsTrx20 _and T55G-K48D_OsTrx20_, 8542.5 M^−1^ cm^−1^ for WtOsTrx23 and G41T_OsTrx23_ and 28545 M^−1^ cm^−1^ for OsNTRB. 

**DTNB assay: **Reduction of WtOsTrx20 and mutants with OsNTR1 was assayed in reaction containing NADPH as electron donor and DTNB (5, 5- dithiobis (2- nitrobenzoic acid) as final substrate [21]. The assay mixture ‎contained 100 mM potassium phosphate pH 7.5, 10 mM EDTA, 0.1 mg/ml bovine serum ‎albumin (BSA), 200 µM DTNB, 200 µM NADPH and 5 µM Trx. The reaction was initiated by addition of 80 nm NTR and the reduction of DTNB was measured by use of a spectrophotometer at 412 nm. The results reflected the formation of 2-nitro-5-thiobenzoic acid (TNB) (ϵ_412nm_= 13,600 M^-1^ cm^-1^) [22]. The reaction mixture without OsTrx was used as a control. All reactions were repeated three times.

**GSH- dependent insulin** **assay: **The reduction of Trxs by GSH was tested in a reaction containing 300 ‎µl reaction mixture containing GSH as electron donor and insulin as final substrate. The reaction mixture contained ‎100 mM potassium phosphate buffer, pH 7,‎ 10 mM EDTA and 1mg/ml insulin and 5 µM OsTrx. The reactions were initiated by addition of 20 mM GSH and monitored spectrophotometrically at 650 nm for 100 min. A reaction mixture without Trx was run as control. All reactions were repeated three times.

**Sequence Analysis: **Multiple alignment between different rice Trx h isoforms was performed using the ClustalW software [http://www.ebi.ac.uk/Tools/msa/clustalw2/].

## RESULTS

**Figure 1:**
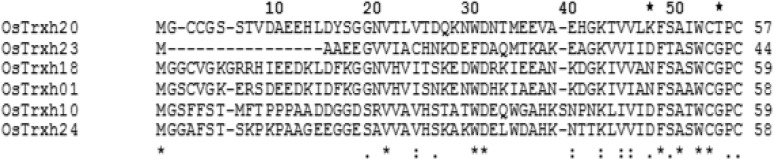
Multiple alignment between different rice Trx h isoforms using the ClustalW software ‎‎(http://www.ebi.ac.uk/Tools/msa/clustalw2

A multiple alignment among the amino acid sequences of rice Trx h isoforms ‎demonstrated that the active site sequences of OsTrx1, OsTrx10, OsTrx15, OsTrx18, ‎OsTrx23, OsTrx24, and OsTrx26 corresponds to classical Trx-active site motif (Trp-Cys-‎Gly -Pro-Cys). In the active site sequence of OsTrx20, however, Gly has been replaced with Thr. Moreover, in other plant Trx h isoforms a conserved Asn/Asp is observed before active ‎site. This amino acid has been replaced by a Lys in OsTrx20 (Fig. 1).

**Figure 2:**
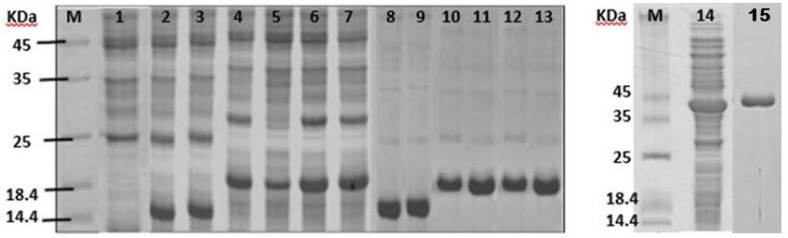
Overexpression of wild types and mutants in *E. coli*. SDS-PAGE analysis for veriﬁcation of expression and puriﬁcation. Total soluble protein extracted from *E.coli* harboring pET-15b (lane 1), pET15b-WtOsTrx23 (lane 2), pET15b-G41T_OsTrx23_ (lane 3), pET15b-Wt_OsTrx20_ (lane 4), pET15b-K48D_OsTrx20 _(lanes 5), pET15b-T55G_OsTrx20_ (lane 6), pET15b-T55G- K48D_OsTrx20_ (lane 7) and OsNTRB (lane 14) 4 h after addition of IPTG. Puriﬁed His-WtOsTrx23 (lane 8), His-G41T_OsTrx23_ (lane 9), His-WtOsTrx20 (lane 10), His-T55G_OsTrx20_ (lane 111), His-K48D_OsTrx20_ (lane 12), His-(T55G)(K48D)_OsTrx20_ (lane 13) and OsNTRB (lane 15

The proteins Wt_OsTrx20_, Wt_OsTrx23_, OsNTRB and mutants K48D_OsTrx20_,T55G_OsTrx20_, T55G-K48D_OsTrx20_ and G41T_OsTrx23_ with a His.tag at the N-terminus were found in the soluble fraction of the *E. coli* transformant culture after induction with IPTG. The theoretical molecular mass of Wt_OsTrx20_, Wt_OsTrx23_, OsNTRB, K48D_OsTrx20_, T55G_OsTrx20,_ T55G-K48D_OsTrx20_ and G41T_OsTrx23_ were 17.5, 15.4, 39.9, 17.5, 17.5, 17.5 and 15.4 KDa respectively. SDS-PAGE of cell extracts revealed a prominent polypeptide band of the expected molecular mass (Fig. 2, lanes 2–7 and 14). The recombinant proteins were puriﬁed from the crude extracts by nickel affinity chromatography (Fig. 2, lanes 8-13 and 15).

**Figure 3:**
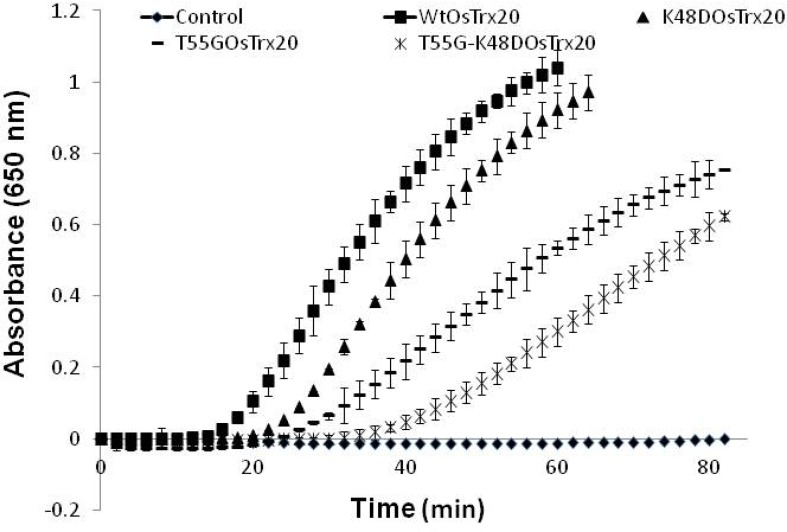
Insulin reduction WtOsTrx20 and mutants in the presence of GSH. The control shows reaction containing GSH and insulin without addition of Trx

The insulin precipitation was monitored by the increase in turbidity at 650 nm (∆OD_650_) in insulin-disulfide reduction assays containing GSH in the presence of WtOsTrx20, T55G_OsTrx20, _K48D_OsTrx20 _or T55G-K48D_OsTrx23 _(Fig. 3). The results showed that GSH actively reduced WtOsTrx20. However, in comparison to WtOsTrx20 the rate of insulin reduction remarkably decreased when T55G_OsTrx20_ and T55G-K48D_OsTrx20_ was used in reaction. In addition, the rate of reaction slightly decreased in the presence of K48D_OsTrx20. _Thease results suggest that Thr55 has critical role in reduction of OsTrx20 by GSH.

**Figure 4:**
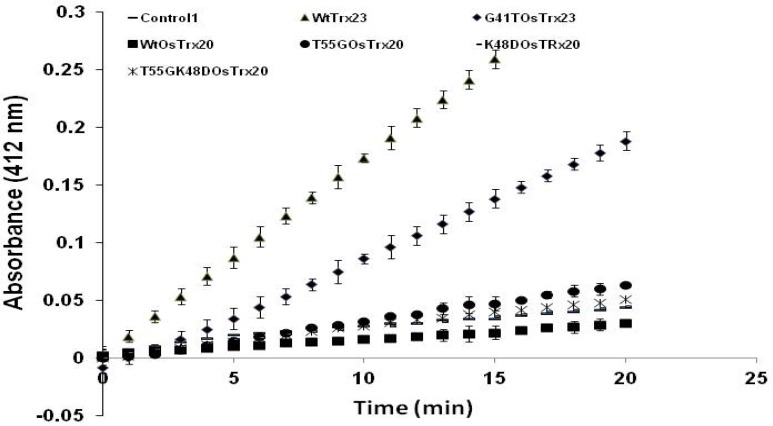
Time course of reduction of Trxs by OsNTRB as monitored by reduction of DTNB. Control shows the time course of DTNB reduction by OsNTRB without addition of OsTrx

The results of DTNB assay showed that recombinant form of OsNTRB was able to actively reduce WtOsTrx23 (Fig. 4). However the initial velocity of reaction was remarkably decreased when the mutant G41T_OsTrx23_ was used instead of WtOsTrx23. In contrast to OsTrx23, the isoform OsTrx20 was not reduced by OsNTRB. However the substitution of Thr 55 with Gly and Lys48 with Asp slightly increased the reaction of this isoform with OsNTRB. These results suggest that the residue Gly41 which is located in the first position after N-terminal active site Cys is critical for NTR-Trx interaction.

## DISCUSSION

It has been very well documented that some members of the Trx h subgroup III characterized in plants with an atypical active site (WCTPC or WCSPC) are not reduced by NADPH/NTR system but they can be reactivated by GSH/Grx system [6, 7]. In contrast to Subgroup III, the members of subgroup I and II with typical WCGPC active site are reduced by cytoplasmic/mitochondrial NTRs [16, 19]. In rice Trx h isoforms can be divided in three subgroups on the basis of their amino acid sequences [19]. Previously we show that OsTrx23, a member of subgroup I is actively reduced by both OsNTRB and OsNTRA, but cannot be reduced by GSH [19]. In contrast, OsTrx20 which belongs to subgroup III and carry an atypical active site can be reactivated by GSH/Grx system [19]. The isoform OsTrx23 and OsTrx20 are predicted to be dominantly localized in cytoplasm and chloroplast, respectively [23]. The isoform OsTrx20 is expressed under light in the leaves of rice seedlings [23]. The activity of OsTrx20 for reduction of target proteins was demonstrated to be pH-dependent and the residues Thr55 and Lys48 were shown as key residues for instability of its activity under pH changes [20]. 

In this work, we showed that the rate of reduction of G41T_OsTrx23_ by OsNTRB decreases in comparison to WtOsTrx23 suggesting the presence of Thr in this position impairs the interaction of OsTrx23 with OsNTRB. In addition we showed that the isoform WtOsTrx20 was actively reduced by GSH and substitution of Thr55 with Gly in OsTrx20 resulted in remarkable decrease of rate of reduction by GSH. These results supports the critical role of residues situated between two active site Cys in redox activity of Trxs [24]. and highlight the role of first residue after N-terminal active site Cys in determination of the system that Trxs can be reduced in. 

Taken together the presence of atypical active site for some h-type Trx isoforms, with Thr or Ser substitution for the largely conserved Gly in the first position after N-terminal active site Cys might be an evolution change that enables these isofroms to be reduced by GSH and adapt them in the cell organelles lacking cytoplasmic/ mitochondrial NTR isoforms.

## Conflicts of Interest:

The authors declared no conflict of interest.
